# Does an active lifestyle matter? A longitudinal study of physical activity and health-related determinants in older adults

**DOI:** 10.3389/fpubh.2022.975608

**Published:** 2022-08-22

**Authors:** Kaja Teraž, Saša Pišot, Boštjan Šimunic, Rado Pišot

**Affiliations:** ^1^Institute for Kinesiology Research, Science and Research Centre, Koper, Slovenia; ^2^Faculty of Sport, University of Ljubljana, Ljubljana, Slovenia

**Keywords:** older adults, aging, physical activity, wellbeing, quality of life, leisure time

## Abstract

**Introduction:**

It is well-known that regular physical activity, and thus an active lifestyle, has positive effects on aging and general wellbeing. However, the question remains as to whether regular or increased physical activity can improve self-perception of health status and quality of life in older adults.

**Methods:**

We conducted a longitudinal study on a group of active older adults between 2013 and 2021. At baseline, i.e., the 1st measurements (baseline), 147 participants were enrolled (mean age 68.4 ± 5.6). After 8 years, in 2021 (follow up), 52 older adults (mean age 75.9 ± 5.3 years) were measured. For the purpose of this study, we included 52 older adults participated at both time-points. For both measurements, participants reported their physical activity and sedentary behavior using the Global physical activity questionnaire (GPAQ), socio-demographic and environmental determinants, recording their self-perception in terms of overall wellbeing. Furthermore, we conducted a qualitative study using semi-structured interviews to obtain subjective data on the changes and events that may have affected physical abilities and general health over an 8-year period.

**Results:**

At the follow up, participants reported lower physical activity and sedentary behavior compared to baseline, but still met health-enhancing physical activity (*HEPA*) standards for total self-reported physical activity (>3,000 METmin/week). In addition, they rated their overall health (*p* < 0.001), physical fitness (*p* < 0.001), psychological wellbeing (*p* < 0.001) and overall quality of life (*p* < 0.001) as better. The qualitative data confirmed that the 8-year period involved changes in physical activity. Specifically, they have continued to carry out physical work (gardening, working in the vineyards, olive groves), but previously organized physical activities were replaced by walks in nature, which probably also influenced the reduction of sedentary behavior.

**Conclusion:**

After 8 years, as expected, participants reported a decrease in physical activity and a lower level of sedentary behavior. It appears from the interview that healthy older adults filled their days with daily tasks and found more time for walking. Individuals who were more active in the past 8 years also reported better overall health and wellbeing. Selected variables correlated with an active lifestyle and better perceptions of quality of life.

## Introduction

Active engagement, absence of disease, and good physical and cognitive function are key components of successful aging (1). Various leisure activities have a positive impact on older adults' perceptions of wellbeing (2–4). It is already known that there is a positive correlation between an active life and life satisfaction (5), and physical activity in particular can have a positive impact on health and functioning of older adults (6–8). Yet, many people are much less active than recommended (9, 10). Most older adults do not engage in enough substantial physical activity (11). Usually their physical activity is represented by housework or going to the supermarket (12), but unfortunately, this is not enough to stay healthy. To maintain protection against the development of age-related functional and health impairments, planned physical activity is necessary (13).

In addition to physical activity (PA), sedentary behavior (SB) also plays an important role in healthy aging. According to the World Health Organization guidelines, the definition of SB is any waking behavior characterized by energy expenditure of 1.5 METs or less while sitting, lying, or leaning (13). But SB, or “too much sitting,” is not the same as physical inactivity or “too little exercise.” Individuals can meet current recommendations for physical activity but, on the other hand, spend the rest of the day heavily sedentary (14). Alternatively, they may not meet current recommendations for moderate to vigorous physical activity (MVPA) but still have very low SB. According to the Survey of Health, Aging, and Retirement in Europe, 11.8% of older adults in Slovenia are physically inactive (11).

High SB and low PA levels are independent risk factors for major chronic diseases (13, 15). According to (16), older adults are aware that any kind of physical activity is necessary for healthy aging. Self-perception of one's health reflects the ability to function in a given social and organizational situation (17). Individuals with low scores of self-perceived health status are more likely to use medical services and have lower functional independence than those with the opposite attitude toward their health (18). It is already known that leisure-time PA is associated with better self-rated health in young adults (19, 20). But what about older adult? After reviewing the literature, we did not find a longitudinal study with a data collection of physical activity and general wellbeing interval of 8 years. Therefore, a longitudinal approach to data collection would be necessary in this area of research to evaluate the importance of active lifestyle on self-awareness of health and wellbeing of older adults. Our research question was whether older adults with an active lifestyle perceive their health status and wellbeing to be better. We wanted to determine whether the influence of an active lifestyle, such as regular or increased PA and low SB, affects self-perception of individual health status and quality of life in older adults.

## Methods

### Participants

This study included baseline data of 52 participants from the Physical Activity and Nutrition for Great Aging (PANGeA) mass measurements in 2013. The baseline study enrolled older adults (≥60 years) from three Slovenian cities – Koper, Ljubljana and Kranj. At baseline, in Koper, we enrolled 147 older adults aged 60–79 years from the Slovenian city of Koper and its surroundings living independently. Inclusion criteria for the first leg were older adults aged between 60 and 80 living independently. Exclusion criteria for baseline measurements were as follows: the inability to walk a distance of 2 km independently and continuously; severe cognitive decline [MoCA score <10 points (after correction for age and schooling)]; acute illness or with a recent hospitalization (in the 6 months prior); having diabetes mellites or insulin therapy or being on medications other than metformin. After 8 years, in 2021, all participants from baseline measurements made in Koper were invited to take follow-up measurements. Fifty-two participants were measured again (22 men and 30 women, mean age: 75.9 ± 5.3 years). For the follow-up, we invited participants by mail and over the phone. Of the participants who were unwilling or unable to respond to the follow-up measurement, we obtain the reason for dropping out (death, unreachable, other health issues). Specific data is described in the “Results” section.

### Ethical approval

The original mass measurements study, PANGeA, which was co-financed by the Cross-border Cooperation Program Slovenia – Italy 2007–2013, was conducted according to the standards set by the latest revision of the Declaration of Helsinki from 2012 to 2014. Both the baseline and follow up measurements were approved by the National Ethical Committee of the Slovenian Ministry of Health (baseline ethical approval no. 102/04/12; follow-up ethical approval no. 0120-76/2021/6) and confirmed by the ZRS Koper Scientific Council no. 0624-77/21. Moreover, the clinical trial protocol was registered on ClinicalTrials.gov, Identifier: NCT04899531. The purposes and objectives of this study were carefully explained to the participants and written informed consent was obtained from all of them.

### Study protocol

The data was collected in Koper on two occasions (baseline and follow-up) at the Institute for Kinesiology research laboratory. Participants came for measurement at a prearranged time and completed a series of tests in this order: (see below).

### Measurements

The PANGeA Questionnaire consists of several different parts covering general health status, wellbeing and lifestyle (PA, nutrition and habits) of the older adult population. It includes GPAQ – Global physical activity questionnaire for self-assessment of PA, and has adapted part of the European Health Interview survey – EHIS (21) to assess eating habits (regular diet, type of diet) and indicators of quality of life.

#### Self-assessment of physical daily habits and general health and wellbeing

To evaluate PA habits and SB, the Global physical activity questionnaire (GPAQ) was translated into Slovene and used (22). It consists of 16 questions divided into three domains as well as a sedentary behavior section. The three domains are as follows: activity at work, travel to and from places on foot, and physical activities. Since the study mainly involved retired elderly people, we included all activities that elderly people have to do at home or around the house (e.g., gardening, working in vineyards, olive groves, etc.) within the scope of occupational activities.

Bearing in mind that quality of life has a frame of reference which is broader than aging, we used a part of EHIS (adapted into Slovenian language in 2007). Self-assessment of general health status, physical condition, psychological wellbeing and general quality of life were assessed through the Likert scale, from 1-poor to 5-excellent. Participants were asked to respond to five categories: (a) general health status, (b) physical condition, (c) psychological wellbeing, (d) general quality of life and (e) extent to which they care for their health (1- do not care at all; 2- care very little/not enough; 3- care somewhat; 4- care quite a lot, 5- care enormously). Participants also had the option of answering “do not know”; these responses were not considered in the statistical analysis of the data.

#### Semi-structured interviews

Additional qualitative material was collected by interviews to cover changes in the daily life of an individual that could affect physical abilities and general health over the period of 8 years. The background topic for nodes and codes was “changes in everyday life” regarding daily practices, routines in *diet, PA domains and general* wellbeing. Participants were invited to participate in the measurements by mail and then again by phone call.

Researchers did not encounter any ethically questionable situations with participants while conducting the interview. Anonymity was guaranteed to all participants, and each respondent was given a code number. All interviewees agreed to participate in the research (each of them signed a consent form) and were informed of all conditions under which data acquired through research would be used. The interviews were recorded using a smart phone application. All recordings are stored in the researchers' private databases and were used solely for transcription purposes.

### Data analysis

Quantitative data from the questionnaire were reported as means (standard deviations) for continuous variables or numbers (percentages) for categorical variables. All statistical analyses were performed using Microsoft Excel 2016 (Microsoft Corporation, Redmond, WA, USA) and SPSS Statistics version 22 (IBM, Chicago IL, USA). Comparison between the sexes and points in time (baseline and follow-up) for normally distributed outcome measures (PA and SB) were performed by means of Mixed model ANOVA, to account for between-subject (differences between women and men) and within-subject (differences between baseline and follow-up measurements) variability. G^*^Power (23) was used for sample size calculation and the detection of a large effect size (i.e., *f* = 0.25) for the group^*^time interaction of the Mixed model ANOVA (two groups, two measurements) was made (α = 0.05, power = 0.95). Outcome measures of general health and wellbeing were not distributed normally and as such we used the Wilcoxon Signed Ranks Test to compare baseline and follow-up outcome measures. Correlation between self-assessed PA and SB and self-assessed general health and wellbeing was evaluated using Spearman's rank correlation coefficient. Statistical significance was set at *p* ≤ 0.05.

For the qualitative analysis, the software NVIVO 12 was used for data storage of transcriptions, and for recording connections, annotations, and codes and nodes layout. Additionally, the data analysis, imaginative exploration, and reflection were carried out by researchers. The interviews were conducted using an agreed protocol under an initial code and set of basic info: date and time of the conducted interview. To capture a personal perspective on the significance of the changes occurring during this period between T1 and T2, we encouraged respondents to answer questions on whether anything had happened in the 8 years since the first measurement that had influenced a change in their daily routines.

We set basic nodes: “changes in daily routines,” “changes in eating habits,” “changes in physical activity/exercise” and “other (new) habits.” A more detailed analysis of the empirical material within the nodes of changes in daily routines due to stressful life events was divided into:

a) health-related issues (illness, injuries) and
b) social issues; negative familial events (death, severe illness/injury) or positive familial events (birth of grandchildren, new relationship, etc.).

Additionally, more specifically we focused on changes of:

a) “Physical activity (PA)” resulting in more sub-entries (less PA, more PA, no changes in PA, new PA program).
b) “Eating habits”: (un)healthier eating habits, eating less/more (quantity), “mindful eating,” no changes in eating habits.
c) any other new habits.

## Results

After 1 month of personal engagement by researchers to persuade participants to re-engage in the study, we got a final sample of 52 participants from the baseline (*n* = 147), which is a fair response rate (35%). Additionally, we obtained different reasons for non-participation in measurements: 13 participants died (information was obtained from the Central population register of the Republic of Slovenia) and an additional 82 participants were unable to participate in follow up measurements due to various reasons, of which 66 participants did not respond to the re-invitation. Eleven participants could not participate due to health problems, while 5 participants made an appointment but did not come to take the measurements. Characteristics of older adults who participated at both stages are described in Tables 1, 2. The mean age of participants who are included in this study, at the baseline was 68.4 ± 5.6 years and the majority were women (57.7 %).

**Table 1:** Description of sex and education frequencies of sample.

**Variables**	**Baseline**	
	***N***	**(%)**
**Sex**
Women	30	57.7
Men	22	42.3
**Education**
Short vocational upper secondary	10	19.2
Technical upper secondary	18	34.6
General upper secondary	1	1.9
Higher vocational education	9	17.3
First cycle academic education	13	25.0
Master/Doctor of science	1	1.9

**Table 2:** Sample description on baseline and follow-up measurements.

**Variables**	**Baseline**		**8-year follow-up**	
	***N***	**(%)**	***N***	**(%)**
**Marital status**
Single	3	5.7	4	7.7
Married/cohabiting	32	60.4	33	63.5
Widowed/divorced/separated	13	24.5	12	23.1
Decline to answer	4	9.4	3	5.7
**Age (years)**
60–64	18	34.6	/	/
65–69	13	24.9	5	9.6
70–74	11	21.1	20	38.5
75–79	10	19.3	10	19.2
80–84	/	/	14	26.9
85–89	/	/	3	5.8
**Number of members sharing the same residence**
Living alone	12	22.6	17	32.7
Living with a partner	28	52.8	27	51.9
Living with children	1	1.9	1	1.9
Living with grandchildren	2	3.8	1	1.9
Living with children and grandchildren	6	11.3	6	11.3
Declined to answer	4	7.5	0	/
Number of comorbidities	3.4	/	3.5	/
Number of prescribed medicines	1.9	/	2.9	/

There was a statistically significant difference in PA and SB between baseline and follow up measurements (see Table 3). Moreover, we found a statistically significant increase in prescribed medications (*t* = −3.252, *p* = 0.002).

**Table 3:** Self-reported physical activity and sedentary behavior (SB) at the baseline and in follow up measurements.

	**Baseline**		**8-year follow-up**		***p*_time_**	*p*_time*sex_
	**Men**	**Women**	**Men**	**Women**		
GPAQ (METmin/week)	5,988 ± 3,008	4,515 ± 3,096	4,425 ± 2,821	3,437 ± 2,504	**0.029**	0.681
SB (min)	244 ± 105	272 ± 122	119 ± 51.6	147 ± 88.6	**<0.001**	0.998

GPAQ – physical activity related energy expenditure from Global Physical Activity Questionnaire (in metabolic equivalent minutes per week). Bold values indicate the statistically significant difference.

Further analysis of self-reported PA (see Table 4) showed a significantly lower amount of moderate work-related PA in follow-up measurements (*F*_(1, 45)_ = 4.972, *p* = 0.031). None of the participants (men or women) reported vigorous PA in their work activities.

**Table 4:** Self-reported physical activity (PA) of different levels of intensity for men and women at the baseline and in follow-up measurements.

	**Baseline**		**8-year follow-up**		***p*_time_**	*p*_time*sex_
	**Men**	**Women**	**Men**	**Women**		
Moderate PA at work (time)	3,014 ± 2,670	2,315 ± 3,060	1,771 ± 1,451	1,431 ± 1,930	**0.031**	0.709
Walking time (min)	529 ± 740	680 ± 835	443.1 ± 491	607 ± 763	0.564	0.965
Vigorous PA (min)	377 ± 854	148 ± 471	228.6 ± 619	46 ± 166	0.219	0.816
Moderate PA (min)	1,657 ± 1,522	1,258 ± 1,077	1,303 ± 1,640	1,250 ± 1,301	0.463	0.481

PA, physical activity. Bold values indicate the statistically significant difference.

After analyzing self-reported general health and wellbeing, we found significantly better general health (*Z* = −4.705, *p* < 0.001), physical condition (*Z* = −4.603, *p* < 0.001), psychological wellbeing (*Z* = −5.489, *p* < 0.001) and general quality of life (*Z* = −5.806, *p* < 0.001) in older adults who participated in the study (Table 5).

**Table 5:** Self-assessment of general health and wellbeing at the baseline and in follow-up measurements.

	**Baseline**	**8-year follow-up**	***p*-Value**
General Health status	2.4 ± 0.6	3.5 ± 0.6	<0.001*
Physical condition	2.4 ± 0.7	3.4 ± 0.6	<0.001*
Psychological wellbeing	2.1 ± 0.7	3.9 ± 0.7	<0.001*
General quality of life	2.2 ± 0.6	4.0 ± 0.5	<0.001*
Concern for health	3.0 ± 0.5	3.1 ± 0.5	0.366

The symbol and bold values indicate the statistically significant difference.

In baseline measurements, there was a positive correlation between total PA and concern for health (*r* = 0.441, *p* = 0.045) and between moderate PA and psychological wellbeing (*r* = 0.627, *p* = 0.002), only for men.

In follow up measurements, there was a positive correlation between walking time and general health status (*r* = 0.472, *p* = 0.027) and between vigorous PA and psychological wellbeing (*r* = 0.465, *p* = 0.029) for men. Meanwhile, in women we found a positive correlation between moderate PA and general health status (*r* = 0.378, *p* = 0.047) and between moderate PA at work and psychological wellbeing (*r* = 0.466, *p* = 0.011).

### Semi-structured interviews

To help us understand the quantitative results of the questionnaire, quantitative research (in the form of semi-structured interviews) was used to explain in more detail why self-assessment improved in all five categories for both men and women (general health, physical condition, psychological wellbeing, general quality of life, and concern for health), as they rated them statistically better after the 8-year period. The only exception was the “concern for health” category, for which there was no statistically significant difference, and which was rated lower by women.

All but one of the participants taking part in the semi-structured interviews (*N* = 51) were men (*N* = 22), and interviews lasted 7.53 min on average. All interviews were recorded on a smartphone, with the personal consent of the participants, and were used for transcription and further qualitative data analysis. The main start question was “if anything had happened to interrupt or change their daily routines in the 8-year period since the first baseline measurement?

Responses were divided at the first level into those who reported no changes and those who perceived changes. Interestingly, one-third of participants [*N* = 17, men (*N*) = 9] reported an unchanged daily routine, which in most cases signifies the absence of a serious illness or injury. Furthermore, in the first phase, we examined those who reported changes [*N* = 31, men (*N*) = 13] within the scope of a broader range of factors associated with aging that may have influenced self-assessment of quality of life, namely:

A) Changes in health status, i.e., those that:
  - observed a decrease in vitality as a natural sign of aging.
  - reported illness.
  - reported injuries.
  - reported new routines.
  - and the influence on PA/exercise habits and eating habits as the most prominent features of a healthy lifestyle.
B) Reported life events (negative or positive) that influenced changes in daily routine.

Table 6 shows health-related changes that interrupted the daily routine of another third of those questioned (17 participants). Only two women noted a decline in vitality, and thus motor skills, as a result of natural aging.

**Table 6:** Changes in daily routines over an 8-year period.

	**Total (** ***N*** **)**		**Men (** ***N*** **)**		**Women (** ***N*** **)**	
No changes to routine	17	33%	9	18%	8	16%
Changes related to COVID-19 measures	2	4%	0	0%	2	4%
Changes to routine	31	61%	13	25%	19	37%
Positive impact
Health-related changes	2	4%	1	2%	1	2%
Positive psychosocial factors	4	8%	2	4%	2	4%
Negative impact
Natural health decline	2	4%	0	0%	2	4%
Health-related illness	7	14%	4	8%	3	6%
Health-related injuries	5	10%	2	4%	3	6%
Injuries and neg. psychosocial factors	3	6%	0	0%	3	6%
Negative psych-social factors	9	18%	4	8%	5	10%
Total	51	100%	22	43%	29	57%

We can see in detail that the presence of diseases was given as a reason for changes in daily routine by seven participants (13.7%; men = 4), who most frequently mentioned strokes, heart attacks, cancer, and respiratory and neurological diseases. The other health factor, injuries, was reported by five participants, and another three participants reported a combination of injuries and negative psychosocial factors, with the most frequently cited injuries being fractures (hand, ankle, hip, and spine injuries).

Interestingly, negative life events, rather than health-related factors, were the most frequently cited reason for interruption of daily routine in nine participants [men (*N*) = 5], with the death of a spouse (*N* = 3), death of a close relative (*N* = 4), and poor relationships (misunderstandings with relatives) being the most frequently mentioned.

When considering PA changes in habits and diet as factors of healthy lifestyles, female participants reported in two cases that changes in daily routines occurred only after COVID-19 measures were implemented.

In addition, in five cases, participants reported that PA decreased during COVID-19 due to the annulment of organized exercise that could not be replaced with individual exercise. Reduction in PA due to a natural decline in physical capacity forced three male participants to give up more intense exercise: cycling and running. The reason for reducing and abandoning more intense forms of PA was generally health problems [illness and injury = 15, men (*N*) = 7]. Interestingly, two women and one man reported an increase in PA due to more regular exercise on their own (as a result of the COVID-19 lockdown) and additional work in an olive grove, while one woman reported a new breathing exercise as more beneficial to her health.

The majority of those who reported changes in daily routine also indicated changes in diet [12 of 17 participants, men (*N*) = 5]. Most did so for health reasons; they tried to eat healthier and eat more vegetables, and some reported eating less meat. Some tried to avoid certain foods that they thought were unhealthy, such as foods high in sugar and white flour. Participants also reported using more herbs, spices, and other supplements.

In addition, positive changes related to healthy living were mentioned, whereby they began to incorporate regular exercise and a healthier diet into their lifestyle due to the onset of chronic non-communicable diseases (diabetes) and/or injuries (back pain).

Finally, positive life events must be mentioned, such as the joy of new grandchildren and great-grandchildren, which gives new meaning and significance to the lives of older people despite the interruption to their daily routines.

## Discussion

The aim of our study was to evaluate the self-assessed general health status and wellbeing of active older adults over an 8-year period.

This study uses an explanatory mixed-method design (24). In the first phase, quantitative results on self-reported PA, SB and quality of life were obtained using a specially-developed questionnaire (PANGeA), aware that some of the key factors determining quality of life in old age involves considerable overlap with the constituents of positive or successful aging (e.g., maintaining independence, social participation, control over one's life, social role functioning, cognitive ability, adaptability, morale, wellbeing, and life satisfaction) (25). As such, quality of life could be defined as a broader concept, evolving from a variety of disciplinary perspectives - mainly sociological, biomedical, psychological, economic, and environmental. To better understand the possible connection between self-assessed quality of life and successful aging, additional qualitative research with semi-structured interviews was carried out with participants. Semi-structured interviews were used to capture events that may have influenced changes in older people's daily practices, particularly dietary and exercise habits, and that consequently influenced the self-assessment of quality of life.

The study showed that after 8 years, one third of the participants did not notice any changes in their lifestyle, so they continued to live active and healthy lives, the other 2/3 reported changes that were mostly negative in terms of motor decline, occurrence of injuries and diseases, and psychosocial factors and a combination of the above, consistent with a statistically significant decline in both PA and SB. This can be explained by the fact that the participants are now less physically active while engaging in less sedentary activities. Some of them pointed out that their motor skills have decreased, while PA has decreased mainly due to injuries and illnesses. At the same time, they fill their days with various household tasks, so they indicate less sedentary activities. Their PA activity consists mainly of walking and gymnastics (yoga, Pilates), but also dancing, while cycling, walking and games with bats and balls are decreasing. Most active time is spent gardening, working in the olive grove, vineyard and orchard, which was also a seasonal and geographical characteristic of the observed cohort.

In general, after 8 years, all participants still exceeded the limit of >3,000 MET/min/week (in average: men = 4,425 ± 2,821 MET; women = 3,437 ± 2,821), which classified them into the health-enhancing PA (HEPA) population, the HEPA active population. The HEPA active population includes individuals who carry out enough PA for a healthy lifestyle. According to the literature, high-intensity PA is linked to an improved sense of wellbeing and satisfaction (26), and low and moderate PA has more positive effects for the physical health and wellbeing of older people (27, 28). This is also in line with our results; participants who reported a greater amount of walking (this can be categorized as low or moderate PA) also reported a better self-assessed general health status. Moreover, older adults who met the vigorous PA and moderate-to-vigorous PA (MVPA) recommendations score higher in positive affect and lower in depressive symptoms (29). In our sample, men who were more active, specifically vigorously and moderately physically active, reported better psychological wellbeing. On the contrary, Carriedo et al. (29) concluded that the recommended 300 min/week of low PA is not enough to experience a positive mood. We can conclude that participants that were included in our study reported a better self-assessment of general health and wellbeing in comparison to baseline measurements (8 years ago). Therefore, self-selected PA and sustainable lifestyle can have an important influence on psychological benefits as we can confirm with the statement of several participants.

- Woman F164, 81 years: “Since I retired, I've been mostly concerned with nutrition because I was exhausted from work…/… Now I've my own way of eating, my own schedule… I put everything on nutrition, on health, because old age can be beautiful too…/… all these years I liked to do sports, I go to the club, and we go on trips with my colleagues.”
- Man 1M220, 83 years: “I'll tell you my motto: A man must be physically active, must find time for a good book and for smart things.”

However, to maximize our health, we also need to reduce the amount of time spent sitting. Sitting time is associated with a higher risk of adverse health outcomes, including cardiovascular disease, type 2 diabetes, cancer and mortality, even after adjustment for moderate-to-vigorous PA (30–34). In 2020, the WHO published, for the first time, an official document (13) describing SB and its negative impact on health. The WHO recommends that older people should sit as little as possible (13). According to Smith et al. (35), older adults whose SB is more than 11 h per day (in comparison to those who sit 4 h or less) have higher odds of sarcopenia and it may affect their health. Moreover, older adults who are more sedentary also have a lower quality of life (36), therefore the importance of SB behavior is clear. Participants in this study reported that, on average, they spend 244–272 min per day sitting, which is 4.1–4.5 h per day. This is still low SB according to the literature (37).

In addition, most participants reported better overall health and wellbeing. This can also be explained by the positive changes mentioned in the structured interviews related to intentions for a healthier lifestyle. Although participants reported lower levels of physical activity, the changes that occurred over the 8 years led them to intend to exercise more regularly, eat healthier, and generally lead healthier lifestyles. We already know that productive activities can have a positive impact on people's perception on life, both physically and sociologically (4, 27, 38). This may also be related to the different correlations found between PA and overall health or wellbeing at baseline and after completion of the study. At baseline, we found only positive correlations between PA and concern for health or wellbeing. In addition, participants felt better after making positive changes because they made conscious efforts to improve their health, mental wellbeing, and wellbeing.

Nevertheless, in 8 years, the number of comorbidities rose from 3.4 to 3.5, and we found an increase in the use of prescribed medicaments indicating declining health status and the onset of diseases that accompany aging. Mijnarends et al. (39) reported that people with more comorbidities were more likely to have dropped out of the study between the baseline and follow-up measurements. We have also recorded 11 participants that could not participate in follow up measurements due to health problems. Moreover, we can assume that participants who did not take part in follow-up measurements could have an important role in concluding the number of obtained comorbidities. We can speculate that the 68 participants who did not respond to the re-invitation and the five participants who made an appointment but did not show up may have also developed comorbidities which can have an important impact study conclusion. Meanwhile, half (*N* = 26) reported some negative changes in daily routines, mostly due to life events, such as illness, and injury, which on the one hand affected the reported lower PA, but on the other generally showed increased commitment to a healthier diet.

Since we are dealing with only a sample of the population of active older adults, it is understandable that even those whose daily routines have changed during these 8 years estimate their general health, physical condition, psychological wellbeing, and general quality of life, better compared to their elderly peers (4). Interestingly, we found a positive response and easier adaptation to the COVID 19 measures, as most of them maintained their daily routines and replaced organized exercise with walking or online exercise. The majority emphasized the advantage of living conditions, as they live in a suburb in a house with a garden, which could also be a factor in assessing quality of life.

- Man 1M011, 73 years: “My wife and I retired to the garden so as not to be confined at home… There we spent the whole day gardening.”

During the pandemic, however, they noticeably missed social contact with family, friends, and especially organized exercise, which was interrupted.

- Woman 1F089, 75 years: “… you can't go out, you can't go to town… I missed the contact with my friends, my children, they were afraid to infect us. I didn't go to town because you couldn't sit on the bench, you were afraid to drink and eat outside in the park.

The only exception is in the category “concern for health,” with no statistically significant difference and a lower rating amongst women, which could be explained by the perception of the involvement of women in caring for others (caring for sick and elderly parents as well as spouses and grandchildren), which increased even more during the pandemic, where there is not enough space left to take care of their own health.

### Limitations

This study was conducted on a sample of older adults who were re-invited to the follow up measurements after 8 years. However, the sample number is relatively small to draw a firm conclusion and the fact that only physically active participants were included in the study may also influence the conclusions. In addition, while the literature demonstrated the acceptable validity and reliability of the selected questionnaires, wellbeing could be better assessed using multi-trait and multi-method scales. PA was evaluated using a questionnaire, which is not as accurate as the use of instrumental measures such as accelerometers. Moreover, the evaluation of SB was made with just one question, which may have led to biased results. The limitations of using questionnaires with a relatively small number of participants were reduced by the addition of qualitative, semi-structured interviews. The narratives about the past years and the data from the questionnaires provided us with valuable information about changes, events that affected daily life, and some lifestyle factors.

## Conclusions

After 8 years, participants reported lower levels of physical activity (mainly at the expense of moderately intense work activity) which is the result of a subjectively perceived decline in motor abilities and the occurrence of injuries or diseases. They reported also on lower levels of sedentary behavior which may be subjectively conditioned by the perception of sitting time. In summary, participants who were more active during this time also reported better general health and wellbeing… In addition, the qualitative interviews indicate participants' awareness of the importance of a healthy lifestyle for healthy aging, as they include PA and care for healthy nutrition as well as maintaining social contacts in their daily lives. Therefore, we can conclude that an active life matters and an active life leads to a better perception of certain areas of life at old age. The analysis of longitudinal data provided important insights into the long-term impact of active lifestyles on the self-awareness of health and wellbeing in older adults.

## Data availability statement

The raw data supporting the conclusions of this article will be made available by the authors, without undue reservation.

## Ethics statement

The studies involving human participants were reviewed and approved by National Ethical Committee of the Slovenian Ministry of Health. The patients/participants provided their written informed consent to participate in this study.

## Author contributions

KT and SP collected and analyzed the data, and wrote the manuscript. KT, SP, BŠ, and RP edited, revised the manuscript, and approved the final version of the manuscript. All authors contributed to the article and approved the submitted version.

## Funding

This study was conducted in the framework of the project PANGeA: CB147 – Physical Activity and Nutrition for Quality Aging, supported by the Cross-border Cooperation Program Slovenia – Italy 2007–2013. The follow-up measurements were financed within ARRS Research Program (P5-0381) Kinesiology for Quality of Life and as well as Slovenian National Project L5-5550 – Development of noninvasive marker for muscle atrophy (grant no. 1000-15-1988), conducted by Institute for Kinesiology Research Science and Research Centre Koper Slovenia.

## Conflict of interest

The authors declare that the research was conducted in the absence of any commercial or financial relationships that could be construed as a potential conflict of interest.

## Publisher's note

All claims expressed in this article are solely those of the authors and do not necessarily represent those of their affiliated organizations, or those of the publisher, the editors and the reviewers. Any product that may be evaluated in this article, or claim that may be made by its manufacturer, is not guaranteed or endorsed by the publisher.
